# Estimating the daily trend in the size of the COVID-19 infected population in Wuhan

**DOI:** 10.1186/s40249-020-00693-4

**Published:** 2020-06-18

**Authors:** Qiu-Shi Lin, Tao-Jun Hu, Xiao-Hua Zhou

**Affiliations:** 1 Beijing International Center for Mathematical Research, Peking University, Beijing, 100871 China; 2 School of Mathematical Sciences, Peking University, Beijing, 100871 China; 3 Department of Biostatistics, School of Public Health, Peking University, Beijing, 100191 China; 4 Center for Statistical Science, Peking University, Beijing, 100871 China

**Keywords:** COVID-19, Wuhan, Daily, Trend, Size, Infection

## Abstract

**Background:**

The outbreak of coronavirus disease 2019 (COVID-19) has become a pandemic causing global health problem. We provide estimates of the daily trend in the size of the epidemic in Wuhan based on detailed information of 10 940 confirmed cases outside Hubei province.

**Methods:**

In this modelling study, we first estimate the epidemic size in Wuhan from 10 January to 5 April 2020 with a newly proposed model, based on the confirmed cases outside Hubei province that left Wuhan by 23 January 2020 retrieved from official websites of provincial and municipal health commissions. Since some confirmed cases have no information on whether they visited Wuhan before, we adjust for these missing values. We then calculate the reporting rate in Wuhan from 20 January to 5 April 2020. Finally, we estimate the date when the first infected case occurred in Wuhan.

**Results:**

We estimate the number of cases that should be reported in Wuhan by 10 January 2020, as 3229 (95% confidence interval [*CI*]: 3139–3321) and 51 273 (95% *CI*: 49 844–52 734) by 5 April 2020. The reporting rate has grown rapidly from 1.5% (95% *CI*: 1.5–1.6%) on 20 January 2020, to 39.1% (95% *CI*: 38.0–40.2%) on 11 February 2020, and increased to 71.4% (95% *CI*: 69.4–73.4%) on 13 February 2020, and reaches 97.6% (95% *CI*: 94.8–100.3%) on 5 April 2020. The date of first infection is estimated as 30 November 2019.

**Conclusions:**

In the early stage of COVID-19 outbreak, the testing capacity of Wuhan was insufficient. Clinical diagnosis could be a good complement to the method of confirmation at that time. The reporting rate is very close to 100% now and there are very few cases since 17 March 2020, which might suggest that Wuhan is able to accommodate all patients and the epidemic has been controlled.

## Background

As of 5 April 2020, the National Health Commission (NHC) of China has confirmed a total of 81 708 cases of COVID-19 in the mainland of China, including 265 severe cases and 3331 deaths. An additional total of 88 suspected cases were reported. Wuhan has 50 008 confirmed cases. The NHC has also received 890 confirmed reports in Hong Kong Special Administrative Region, 44 in Macau Special Administrative Region, and 363 in Taiwan [1]. More than one million cases have been detected outside China.

Despite the considerable medical resources and personnel that have been dispensed to combat COVID-19 in Hubei province, hospital capacity was overburdened in the early stage of this epidemic. There was a shortage of hospital beds needed to accommodate the rising number of COVID-19 patients. In response to this growing crisis, Wuhan transformed hotels, venues, training centers and college dorms into quarantine and treatment centers for COVID-19 patients. Further, 13 temporary treatment centers were built to provide over 10 000 beds [2]. Therefore, a careful and precise understanding of the potential number of cases in Wuhan is crucial for the prevention and control of the COVID-19 outbreak. Wu et al. [3] provided an estimate of the total number of cases of COVID-19 in Wuhan, using the number of cases exported from Wuhan to cities outside the mainland of China. However, since the number of cases is small, their estimate of the size of the epidemic in Wuhan may not be precise and has large variability. Using the number of cases exported from Wuhan to all cities, including cities in China outside Hubei province, You et al. [4] proposed a method to estimate the total number of cases of COVID-19 in Wuhan. However, their method can only give an estimate of the cumulative number of cases until a certain date.

In this article, we propose a new statistical method to estimate daily number of cases in Wuhan under a similar dynamic equation model as the one in reference [3]. Unlike the one in reference [3], our method can also handle the missing information on whether a case is exported from Wuhan.

## Methods

The spread of COVID-19 outside Hubei province is relatively controlled given the adequate medical resources. We use the reported number outside Hubei as it is a fairly accurate representation of the actual epidemic situation. In this modelling study, we first estimate the epidemic size in Wuhan from 10 January to 5 April 2020, based on the confirmed cases outside Hubei province that left Wuhan by 23 January 2020. Since some confirmed cases have no information on whether they visited Wuhan before, we adjust the number of imported cases after taking these missing values into account. We then calculate the reporting rate in Wuhan from 20 January to 5 April 2020. Finally, we estimate the date when the first patient was infected.

### Data

Data retrieved from publicly available records from provincial and municipal health commissions in China and ministries of health in other countries include detailed information for 10 940 confirmed cases outside Hubei province. An additional table in the Supplementary Materials shows these websites in more detail [see Data_source.xlsx]. Information on confirmed cases including region, gender, age, date of symptom onset, date of confirmation, history of travel or residency in Wuhan, and date of departure from Wuhan. We display demographic characteristics of these patients in Table 1. Among the 7500 patients with gender data, 3509 (46.8%) are female. The mean age of patients is 44.48 and the median age is 44. The youngest confirmed patient outside Hubei province was only 5 days old while the oldest is 97 years old (see Table 1).

**Table 1:** Demographic characteristics of patients with COVID-19 outside Hubei province

Age group (years)	Female (*n* = 3509)	Male (*n* = 3991)	No information (*n* = 3440)
0–20	97 (3)^a^	149 (4)	2
20–39	1076 (33)	1348 (36)	41
40–59	1425 (43)	1598 (43)	38
60–79	630 (19)	578 (15)	39
≥ 80	66 (2)	60 (2)	7
No information	215	258	3313

^a^ Number (%). The percentages do not take missing data into account

We display the epidemiological data categorized by the date of confirmation in Table 2. An imported case means a patient that had been to Wuhan and was detected outside Hubei province. A local case means a confirmed case that had not been to Wuhan. Among the total of 10 940 cases, 6903 (63.1%) have such epidemiological information. The number of imported cases reached its peak on 29 January 2020, and the fourth column of Table 2 shows that the proportion of imported cases declines over time. This might reflect the effect of containment measures taken in Hubei province to control the COVID-19 outbreak [5]. Meanwhile, the daily counts of local cases are over 300 from 2 February to 7 February 2020, which indicate that infections among local residents should be a major concern for authorities outside Hubei province.

**Table 2:** Patient data categorized by the date of confirmation

Date of confirmation	Imported cases (*n* = 3039)	Local Cases (*n* = 3864)	Proportion^a^	No information (*n* = 4037)	Total cases (*n* = 10 940)	Onset to detection (days)
≤ 20 Jan^b^	23	1	96%	2	26	8.83
21 Jan	35	3	92%	7	45	8.60
22 Jan	55	6	90%	16	77	6.11
23 Jan	117	15	89%	52	184	5.37
24 Jan	165	18	90%	92	275	4.56
25 Jan	210	38	85%	110	358	4.15
26 Jan	198	49	80%	163	410	4.05
27 Jan	190	85	69%	208	483	5.00
28 Jan	283	104	73%	252	639	5.58
29 Jan	299	144	67%	280	723	5.43
30 Jan	291	214	58%	260	765	5.13
31 Jan	256	239	52%	308	803	5.05
1 Feb	160	252	39%	266	678	4.83
2 Feb	159	310	34%	269	738	5.55
3 Feb	164	410	29%	322	896	5.76
4 Feb	119	336	26%	294	749	5.99
5 Feb	107	376	22%	266	749	6.10
6 Feb	83	380	18%	258	721	5.92
7 Feb	53	363	13%	201	617	6.17
8 Feb	42	286	13%	202	530	6.49
9 Feb	30	235	11%	209	474	6.66

^a^ The proportion is the number of imported cases divided by the sum of imported and local cases

^b^ The count and average on the first row are taken over all cases confirmed by January 20, 2020

The last column of Table 2 lists the mean time from symptom onset to confirmation for patients confirmed on each day. The median duration of all cases is 5 days, and the mean is 5.54 days. In general, the detection period decreased in the first week after 20 January 2020, but increased since then. The improvements in detection speed and capacity might cause the initial decline, and the rise may be due to more thorough screening, leading to the detection of patients with mild symptoms who would otherwise not go to the hospitals [6].

### Assumptions

The proposed method relies on the following assumptions:

1. Between 10 January and 23 January 2020, the average daily proportion of departing from Wuhan is *p*.
2. There is a *d* = *d*_1_ + *d*_2_-day window between infection and detection, including a *d*_1_-day incubation period and a *d*_2_-day delay from symptom onset to detection.
3. Patients are not able to travel *d* days after infection.
4. The proportion of imported cases in the patients with no information is the same as the observed proportion on each day.
5. Trip durations are long enough that a traveling patient infected in Wuhan will develop symptoms and be detected in other places rather than after returning to Wuhan.
6. All travelers leaving Wuhan, including transfer passengers, have the same risk of infection as local residents.
7. Traveling is independent of the exposure risk to COVID-19 or of infection status.
8. Recoveries are not considered in this method.

Assumptions 1–4 are used explicitly in the Methods section. They are fundamental assumptions for our statistical model. Other assumptions might also affect the result of our model, and we make some remarks about our assumptions.

- 10 January 2020 is the start of Chinese New Year travel rush, and 23 January 2020, is the date of Wuhan lockdown [5]. In the total of 10 940 cases, only 131 (1.2%) cases’ date of departure from Wuhan are not in this period. They are excluded from our analysis.
- If the true average daily proportion of leaving Wuhan is larger than the assumed *p*, this violation of Assumption 1 could lead to overestimation of the number of cases in Wuhan.
- If the average time from infection to detection is longer than the assumed *d* days, this violation of Assumption 2 would lead to an overestimation.
- If travelers have a lower risk of infection than residents in Wuhan, this violation of Assumption 6 would cause an underestimation.
- If infected individuals are less likely to travel due to the health conditions, this violation of Assumption 7 would cause an underestimation.

In the Supplementary Appendix A, we perform the sensitivity analysis on the effect of some of the violations on our results.

### Notations

Let Day *t*_0_ denote the date of infection for the very first case. Let *N*_*t*_ be the cumulative number of cases that should be confirmed in Wuhan by Day *t*. Other notations of our model are defined in Table 3.

**Table 3:** Notations for our model

Notation	Meaning	State	Reference
*T*_*t*_	The reported number of confirmed cases outside Hubei province on Day *t*	Known	Official websites
*I*_*t*_	The observed number of imported cases on Day *t*	Known	Official websites
*L*_*t*_	The observed number of local cases on Day *t*	Known	Official websites
*U*_*t*_	The number of cases with no information on Day *t*	Known	*U*_*t*_ = *T*_*t*_ − *I*_*t*_ − *L*_*t*_
*x*_*t*_	The number of imported cases on Day *t*	Estimated	
*X*_*t*_	The cumulative number of imported cases by Day *t*	Estimated	${X}_t={\sum}_{k=1}^t{x}_k$
*N*_*t*_	The cumulative number of cases that should be confirmed in Wuhan by Day *t*	Estimated	
*t*_0_	The date of infection for the very first case	Estimated	
*p*	The daily probability of departing from Wuhan	Known	[7, 8]
*d*	The time from infection to detection	Known	[9]
*t*_*c*_	The time when *N*_*t*_ increases at the fastest rate	Known	From our data
*r*	A parameter that determines the growth rate of *N*_*t*_	Known	[3]
*K*	The size of the population that are susceptible to COVID-19 in Wuhan.	Estimated	

The numbers *T*_*t*_, *I*_*t*_, and *L*_*t*_ are the observed data used in our model, *t*_*c*_, *r*, and *K* are the parameters that determine how *N*_*t*_ changes over time.

### Model

The growth trend of the size *N*_*t*_ of infected population is determined by the following ordinary differential equation:

1 $$ \frac{d{N}_t}{dt}=\frac{r}{K}{N}_t\left(K-{N}_t\right),\kern0.5em r>0,K>0, $$

where *K* is the size of the population that are susceptible to COVID-19 in Wuhan, and *r* is a constant that controls the growth rate of *N*_*t*_. This is the modified version of the famous SIR model [3, 10] in epidemiology. In the equation (1), the growth rate of *N*_*t*_ is proportional to the product of *N*_*t*_ and the number *K* − *N*_*t*_ of people that are susceptible but not infected yet. It is a reasonable model for the epidemic transmission. At the beginning of this epidemic, when *N*_*t*_ is small, people have little knowledge of COVID-19, *N*_*t*_ grows at an exponential rate *r*. As *N*_*t*_ becomes larger, containment measures are taken to control it, the growth rate of *N*_*t*_ slows down, resulting in a sigmoid curve of *N*_*t*_. Detailed explanations of the model (1) are given in the Supplementary Appendix B. The model (1) has an analytical solution,

2 $$ {N}_t=\frac{K}{1+{e}^{-r\left(t-{t}_c\right)}}=K{f}_t, $$

where ${f}_t=\frac{1}{1+{e}^{-r\left(t-{t}_c\right)}}$, and the derivative $\frac{d{N}_t}{dt}$ is maximized at *t* = *t*_*c*_, $\frac{r}{2}=\frac{d\log {N}_{t_c}}{dt}$ is the growth rate of log*N*_*t*_ at time *t*_*c*_, *K* is a parameter to be estimated.

### Estimation

We use data on the confirmed cases who left Wuhan between 10 January and 23 January 2020, to estimate *K*. Under Assumption 2, cases infected on Day *t* will be detected on Day *t* + *d*, so the number of infected cases in Wuhan is *N*_*t* + *d*_ on Day *t*. If *t*_0_ ≤ *t* ≤ *t*_0_ + *d*, there should be no confirmed cases. If *t*_0_ + *d* < *t* ≤ *t*_0_ + 2*d*, imported cases on Day *t* are infected in Wuhan on Day *t* − *d*. There are *N*_*t*_ infected cases in Wuhan on Day *t* − *d*, hence the number of imported cases *x*_*t*_ on Day *t* follows a binomial (*N*_*t*_,  *p*) distribution, where *p* is the assumed average daily probability of leaving Wuhan between 10 January and 23 January 2020. If *t* > *t*_0_ + 2*d*, under Assumption 3, *N*_*t* − *d*_ patients are not able to travel, *x*_*t*_ has a binomial (*N*_*t*_ − *N*_*t* − *d*_,  *p*) distribution. Let *X*_*t*_ be the cumulative number of imported cases by Day *t*, then

3 $$ {X}_t=\sum \limits_{k=1}^t{x}_k\sim \mathrm{Binomial}\left(\sum \limits_{k=t-d+1}^t{N}_k,p\right),\kern0.75em t\ge {t}_0+2d. $$

From equations (2) and (3), ${X}_t\sim \mathrm{Binomial}\left(K\sum \limits_{k=t-d+1}^t{f}_k,p\right)$. The parameter estimate $\hat{K}$ is derived by maximizing the likelihood function

4 $$ l(K)=\left(\genfrac{}{}{0pt}{}{K\sum \limits_{k=t-d+1}^t{f}_k}{X_t}\right){p}^{X_t}{\left(1-p\right)}^{K\sum \limits_{k=t-d+1}^t{f}_k-{X}_t}. $$

The lower and upper bound of the 95% confidence interval $\left[\hat{K_l}\hat{,{K}_u}\right]$ are values such that the cumulative distribution function $F(K)={\sum}_{x=0}^{X_t}l(K)$ equals to 0.975 and 0.025, respectively. The reporting rate is the reported cumulative number of cases in Wuhan on Day *t* divided by our estimated number $\hat{N_t}$. The estimate of the date *t*_0_ of first infection is obtained by solving the equation ${N}_{t_0}=1.$

Determining the number of imported cases *x*_*t*_ plays a crucial role in the modeling procedure. Note that not all cases have clear records on the history of travel or residency in Wuhan, we need to impute the missing values. Under Assumption 4, the proportion of imported cases in the *U*_*t*_ patients with no information is the same as the observed proportion $\frac{I_k}{I_k+{L}_k}$. Therefore,

5 $$ {x}_t={I}_t+{U}_t\times \frac{I_k}{I_k+{L}_k}={T}_k\times \frac{I_k}{I_k+{L}_k}. $$

The average daily proportion of leaving Wuhan between 10 January and 23 January 2020 is estimated to be the ratio of daily volume of travelers to the population of Wuhan (14 million). More than 5 million people were estimated to leave Wuhan due to the Spring Festival and epidemic [7]. This number is mentioned by Wuhan Mayor in a press conference. We assume these passengers left Wuhan between the start of Chinese New Year travel rush on 10 January 2020, and the lockdown of Wuhan city on 23 January 2020. During the travel rush, 34% of the passengers traveled across 300 km [8]. Major cities outside Hubei province are generally over 300 km from Wuhan. This would imply, on average, the daily probability *p* of traveling from Wuhan to places outside Hubei province would be 5 × 0.34/14/14 = 0.009. Li et al. estimated that the mean incubation period of 425 patients with COVID-19 was 5.2 days (95% *CI*: 4.1–7.0) [9]. The mean time from symptom onset to detection calculated from our data is 5.54 days, so we choose *d* = *d*_1_ + *d*_2_ = 11 days. On 29 January 2020, there was the maximum count of imported cases. Since *x*_*t*_ has a binomial (*N*_*t*_ − *N*_*t* − *d*_, *p*) distribution with constant *p*, *N*_*t*_ − *N*_*t* − *d*_ also reaches its maximum at *t*= 29 January 2020. From the logistic function (2), *t*_*c*_ is the midpoint of *t* and *t* − *d*, that is $t-\frac{d}{2}=$ 24 January 2020, which is shortly after the lockdown of Wuhan city [5]. Wu et al. estimated the epidemic doubling time as 6.4 days (95% *CI*: 5.8–7.1) as of 25 January 2020 [3]. From this result, we estimate that $\frac{r}{2}=\frac{d\log {N}_{t_c}}{dt}=\frac{\ln 2}{6.4}=0.1$. Using these values for parameters *p*, *d*, *t*_*c*_, and *r*, we can derive the maximum likelihood estimate $\hat{K}=51\ 273,$ with 95% *CI*: 49 844–52 734.

## Results

**Fig. 1:**
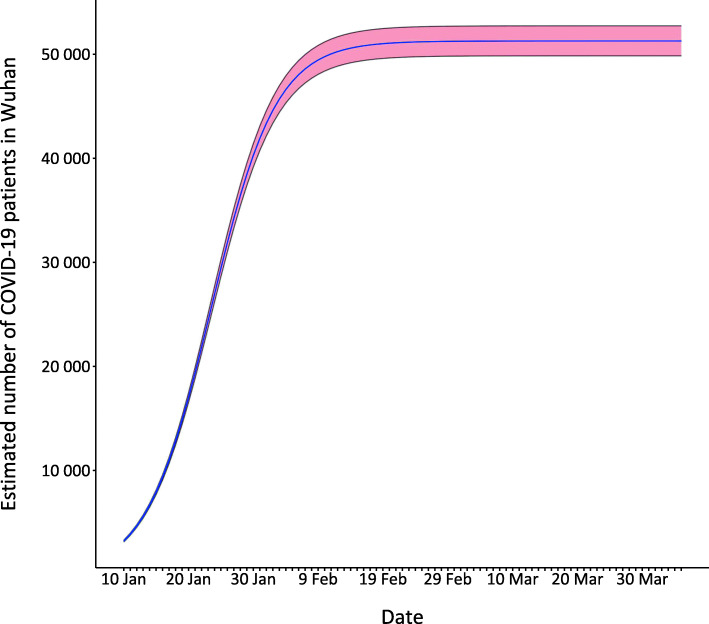
Estimated number of total cases in Wuhan

We estimate the number of cases that should be reported in Wuhan by 10 January 2020, as 3229 (95% *CI*: 3139–3321) and 51 273 (95% *CI*: 49 844–52 734) by 5 April 2020. Figure 1 shows how the estimated number of cases in Wuhan increases over time, together with the 95% confidence bands.

**Fig. 2:**
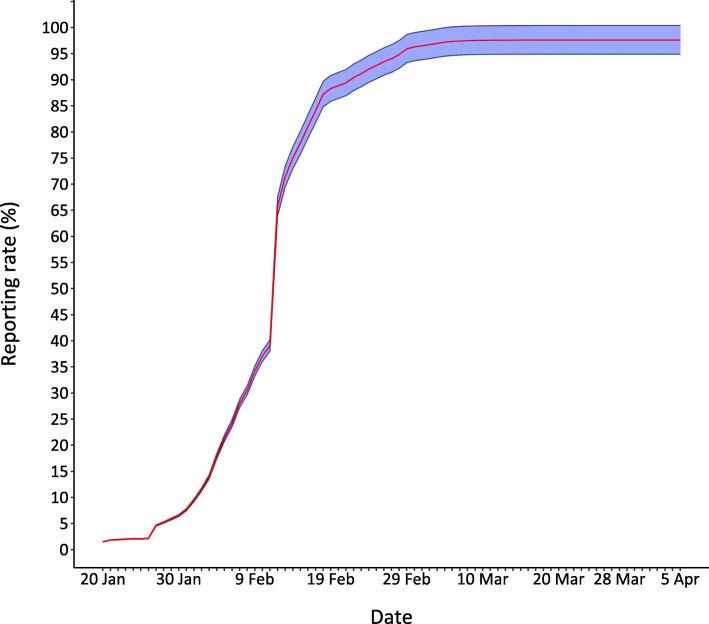
The ratio of reported number of cases to the estimated number

As shown in Fig. 2, the reporting rate has grown rapidly from 1.5% (95% *CI*: 1.5–1.6%) on 20 January 2020 to 39.1% (95% *CI*: 38.0–40.2%) on 11 February 2020. It becomes 71.4% (95% *CI*: 69.4–73.4%) on 13 February 2020, and reaches 97.5% (95% *CI*: 94.8–100.3%) on 5 April 2020.

Table 4 gives the number of confirmed cases reported by Wuhan Health Commission, the estimated number and the reporting rate, as well as the 95% confidence intervals. By solving for *t* in the equation *N*_*t*_ = 1 with the expression of *N*_*t*_ given in (2), we obtain an estimate of the date of first infection as 30 November 2019.

**Table 4:** Model estimated number of cases and reporting rates

Date	Reported	Estimated number (95% *CI*)	Reporting rate (95% *CI*)
20 Jan	258	17 013 (16 539–17 498)	1.5% (1.5–1.6%)
25 Jan	618	29 453 (28 633–30 293)	2.1% (2.0–2.2%)
30 Jan	2639	40 292 (39 169–41 440)	6.6% (6.4–6.7%)
4 Feb	8351	46 601 (45 302–47 929)	17.9% (17.4–18.4%)
9 Feb	16 902	49 449 (48 071–50 858)	34.2% (33.2–35.2%)
11 Feb	19 558	50 036 (48 641–51 462)	39.1% (38.0–40.2%)
13 Feb	35 991	50 437 (49 031–51 874)	71.4% (69.4–73.4%)
18 Feb	44 412	50 962 (49 542–52 414)	87.2% (84.7–89.7%)
23 Feb	46 607	51 158 (49 732–52 616)	91.1% (88.6–93.7%)
28 Feb	48 557	51 231 (49 803–52 691)	94.8% (92.2–97.5%)
4 Mar	49 671	51 257 (49 829–52 718)	96.9% (94.2–99.7%)
9 Mar	49 965	51 267 (49 838–52 728)	97.5% (94.8–100.3%)
17 Mar	50 005	51 272 (49 843–52 733)	97.5% (94.8–100.3%)
25 Mar	50 006	51 273 (49 844–52 734)	97.5% (94.8–100.3%)
5 Apr	50 008	51 273 (49 844–52 734)	97.5% (94.8–100.3%)

## Discussion

Most studies estimating the epidemic size of COVID-19 in Wuhan use the reported number of cases to predict the future trend. These researches ignore the possibility of considerable number of unreported cases in the early stage of this outbreak in Wuhan. We estimate the actual size of epidemic in Wuhan and predict the future trend based on information about COVID-19 cases outside Hubei province. Several recent studies share similar ideas that utilize external data to infer the number of cases in Wuhan. You et al. [4] estimated a total of 3933 cases of COVID-19 in Wuhan (95% *CI*: 3454–4450) that had an onset of symptoms by 19 January 2020. Wu et al. [3] estimated that 75 815 individuals (95% *CI*: 37 304–130 330) have been infected in Wuhan as of 25 January 2020. This number far exceeds 50 008 cumulative cases reported in Wuhan, which seems not very reasonable. Nishiura et al. [11] estimated a total of 20 767 infected individuals as of 29 January 2020 based on a binomial model, which is simplified version of model (3), and eight confirmed cases on three chartered flights evacuating Japanese citizens from Wuhan. These results are estimates of the cumulative number of cases in Wuhan until a certain date and have wide confidence intervals due to limited data size. Using information of over 10 000 confirmed cases outside Hubei province, our statistical method can handle the problem of missing data and estimate the daily number of cases in Wuhan as shown in Fig. 1. Maugeri et al. [12] estimate a total of 8724 (95% *CI:* 8478–8921) infected cases and 92.9% (95% *CI:* 92.5–93.1%) unreported by 23 January 2020 with a proposed SEIRD model based on the reported number of deaths between 23 January and 9 February 2020. However, a total of 1290 cases were added to the death toll in Wuhan on 18 April 2020 by Wuhan government [13]. Thus, the number of deaths used in their research might not be accurate enough, leading to biases in their estimation. In the early stage of this epidemic, estimated numbers given by our method and existing researches are substantially larger than the reported number of confirmed cases. As of 5 April 2020, the reported cumulative number of cases in Wuhan is very close to the estimated number of our model, indicating the effectiveness of our method for long-term epidemic trend prediction. This method can effectively and accurately estimate the actual number of cases when the testing capability is insufficient. Similar statistical methods and ideas can be applied to other countries or regions that are still suffering from the outbreak of COVID-19 to support the prevention and control of this pandemic.

The major limitation of our methodology, as well as many other existing researches, is that time-varying parameters are not taken into consideration. Assumption 1 assumes that the daily probability of leaving Wuhan between 10 January and 23 January 2020, is approximately constant. Our estimate of traveling probability *p* might not be accurate due to the missing of exact daily number of traveling people from Wuhan to places outside Hubei province. We will try to improve the accuracy of *p* with more credible and precise transportation data in future research. Quarantine measures may have influences on some parameters in the epidemiological dynamic model (1), so that these parameters may change over time. It is a future research topic to allow time-varying parameters.

## Conclusions

We provide a computationally efficient method of estimating the daily development of COVID-19 epidemic in Wuhan. The date of first infection is estimated as 30 November 2019. With the introduction of clinical diagnosis in the confirmation of COVID-19 in Wuhan, the reporting rate increases rapidly from about 40% to over 70% in only 2 days in February 2020. Clinical diagnosis could be a good complement to the method of confirmation in the early stage. The suspected cases in Wuhan declined to zero on 17 March 2020. Both the reported and estimated numbers show that there are very few cases since then. This might suggest the epidemic in Wuhan has been under control. The reporting rate is always increasing during this epidemic. As of 5 April 2020, the reporting rate is very close to 100%. Although the medical resources and testing capacity of Wuhan were insufficient at the beginning of this outbreak, Wuhan is now able to accommodate all patients with the assistance from the whole country and effective measures taken in the fight against COVID-19.

## Supplementary information

**Additional file 1.** The first file’s name is Data_source.xlsx. It is a table that lists the official COVID-19 websites of provincial and municipal health commissions for every city in China. It includes province, city and source websites in both Chinese and English. Information of COVID-19 patients are collected from these websites.

**Additional file 2.** The second file’s name is Appendix.docx. It contains two appendices with additional sensitivity analysis and detailed description of our method. They are only for reviewers’ convenience, not for publication.

## Data Availability

All data and materials used in this work were publicly available.
