# Supplementary material for: Estimating the daily trend in the size of the COVID-19 infected population in Wuhan
Source: Infect Dis Poverty. 2020 Jun 18;9:69. doi: 10.1186/s40249-020-00693-4 (PMC7301350; doi:10.1186/s40249-020-00693-4)
Supplement: Supplementary file 2 — Additional file 2. The second file’s name is Appendix.docx. It contains two appendices with additional sensitivity analysis and detailed description of our method. They are only for reviewers’ convenience, not for publication. [file 40249_2020_693_MOESM2_ESM.docx]

**Supplementary Materials to**

**“Estimating the daily trend in the size of the COVID-19**

**infected population in Wuhan”**

The supplement contains two appendices with additional sensitivity analysis and detailed description of our method. They are only for reviewers’ convenience, not for publication. This material will be made available online for all readers.

**Appendix A: Sensitivity analysis**

We explore the sensitivity of the estimate of total cases in Wuhan to our assumptions and choices of parameters $p,d,$ and $r,$ where $p$ is the probability of departing from Wuhan, $d$ is the time from infection to detection, $r$ is a parameter that determines the growth rate of infection. Compared to the baseline, the parameters are chosen under Latin hypercube sampling to reflect the uncertainty, assuming the lower bound and upper bound for each parameter being expanded or shrunk by about 30%. Table S1 summaries the estimate of the number of cases should be reported on 10 January and 5 April 2020 under baseline assumptions and alternative scenarios. Confidence intervals are omitted.

Table S1. Estimated case numbers on 10 January and 5 April 2020, based on different choices of parameters.

|  | $p$ | $d=d_{1}+d_{2}$ | $r$ | 10 Jan | 5 Apr |
| --- | --- | --- | --- | --- | --- |
| Baseline | 0.009 | 11 | 0.2 | 3229 | 51 273 |
| Scenario 1 | 0.007 | 8 | 0.18 | 6410 | 101 796 |
| Scenario 2 | 0.007 | 13 | 0.16 | 6975 | 58 513 |
| Scenario 3 | 0.01 | 14 | 0.2 | 3011 | 36 199 |
| Scenario 4 | 0.011 | 12 | 0.18 | 3511 | 39 962 |
| Scenario 5 | 0.007 | 9 | 0.15 | 9430 | 92 433 |
| Scenario 6 | 0.006 | 12 | 0.23 | 3368 | 70 337 |
| Scenario 7 | 0.007 | 11 | 0.2 | 4290 | 68 119 |
| Scenario 8 | 0.006 | 12 | 0.22 | 3834 | 70 790 |

**Appendix B: Detailed description of the model**

The SIR model consists of three parts – S for the number of susceptible, I for the number of infected, R for the number of recovered.

$$\frac{dS_{t}}{dt}=-\beta I_{t}S_{t}, \frac{dI_{t}}{dt}=\beta I_{t}S_{t}-\gamma I_{t}, \frac{dR_{t}}{dt}=\gamma I_{t}.$$

Adding three differential equations together, we get $\frac{d}{dt}\left( S_{t}+I_{t}+R_{t} \right)=0$, i.e., the sum of three parts is a constant. Assumption 8 implies that the parameter $\gamma$ is zero such that $R_{t}$ is also a constant. Hence, $S_{t}+I_{t}$ is constant too. Denote the sum as ${K\triangleq S}_{t}+I_{t}$. Reparametrize $\beta$ as $\frac{r}{K}$, and use a different notation $N_{t}$ for $I_{t}$, we obtain the model (1).
